# Melatonin Counteracts at a Transcriptional Level the Inflammatory and Apoptotic Response Secondary to Ischemic Brain Injury Induced by Middle Cerebral Artery Blockade in Aging Rats

**DOI:** 10.1089/biores.2015.0032

**Published:** 2015-10-01

**Authors:** Sergio D. Paredes, Lisa Rancan, Roman Kireev, Alberto González, Pedro Louzao, Pablo González, Cruz Rodríguez-Bobada, Cruz García, Elena Vara, Jesús A.F. Tresguerres

**Affiliations:** ^1^ Department of Physiology, School of Medicine, Complutense University of Madrid, Madrid, Spain.; ^2^ Department of Biochemistry and Molecular Biology III, School of Medicine, Complutense University of Madrid, Madrid, Spain.; ^3^ Instituto de Investigación Biomédica de Vigo (IBIV), Xerencia de Xestión Integrada de Vigo, SERGAS, Biomedical Research Unit, Hospital Rebullón (CHUVI), Vigo, Spain.; ^4^ Experimental Medicine and Surgery Unit, Hospital Clínico San Carlos, Madrid, Spain.

**Keywords:** aging, brain, ischemia, melatonin, middle cerebral artery blockade

## Abstract

Aging increases oxidative stress and inflammation. Melatonin counteracts inflammation and apoptosis. This study investigated the possible protective effect of melatonin on the inflammatory and apoptotic response secondary to ischemia induced by blockade of the right middle cerebral artery (MCA) in aging male Wistar rats. Animals were subjected to MCA obstruction. After 24 h or 7 days of procedure, 14-month-old nontreated and treated rats with a daily dose of 10 mg/kg melatonin were sacrificed and right and left hippocampus and cortex were collected. Rats aged 2 and 6 months, respectively, were subjected to the same brain injury protocol, but they were not treated with melatonin. mRNA expression of interleukin-1 beta (IL-1β), tumor necrosis factor alpha (TNF-α), Bcl-2-associated death promoter (BAD), Bcl-2-associated X protein (BAX), glial fibrillary acidic protein (GFAP), B-cell lymphoma 2 (Bcl-2), and sirtuin 1 was measured by reverse transcription–polymerase chain reaction. In nontreated animals, a significant time-dependent increase in IL-1β, TNF-α, BAD, and BAX was observed in the ischemic area of both hippocampus and cortex, and to a lesser extent in the contralateral hemisphere. Hippocampal GFAP was also significantly elevated, while Bcl-2 and sirtuin 1 decreased significantly in response to ischemia. Aging aggravated these changes. Melatonin administration was able to reverse significantly these alterations. In conclusion, melatonin may ameliorate the age-dependent inflammatory and apoptotic response secondary to ischemic cerebral injury.

## Introduction

Stroke is a cerebrovascular accident or brain attack that represents a major cause of death and long-term disability throughout the world. The majority (around 85%) of cerebral strokes are ischemic in nature and result from the occlusion of a major cerebral artery by a thrombus or an embolism. Of these, ∼65% result from vascular occlusion within the territory of the middle cerebral artery (MCA), leading to loss of blood flow and subsequent tissue death in the affected region.^1^

High blood pressure, atherosclerosis or other cardiovascular disorders, history of smoking, obesity, diabetes mellitus, or lack of physical activity are among the risk factors or predisposing conditions for stroke injury to the central nervous system.^2^ Age is, however, the most important independent risk factor, with stroke rates doubling every decade after the age of 55.^3^

The total incidence of stroke is projected to rise substantially over the next years as a result of the expanding elderly population, and it is predicted that stroke will account for over 6% of the total burden of illness by 2020.^4^ Nonetheless, the elderly generally have been underrepresented in clinical trials, creating many uncertainties and less optimal medical care for this group of patients.^5^ This is also related to the very few experimental studies that have been performed in aged animals despite age being considered one of the most significant prognostic markers for poor stroke outcome.

Overproduction of free radicals during cerebral stroke, also referred to as ischemia and reperfusion (I/R), among other pathophysiological mechanisms, including the occurrence of apoptosis in the surrounding tissue of the lesion, are known to contribute to neuronal functional disruption and death.^6,7^ As a result, attention has been paid to melatonin as a neuroprotective drug against I/R brain injury in view of its antioxidant actions opposite to the harmful cellular influence of oxidative stress and proapoptotic factors.^8,9^

Melatonin, a ubiquitously acting indoleamine with direct scavenging actions against free radicals and related products, as well as indirect anti-inflammatory actions,^10–12^ has been shown to attenuate tissue loss and the consequential neurophysiological deficits associated with the transitory interruption of blood supply to the brain, minimizing the neural damage to both gray and white matter resulting from transitory hypoxia followed by reoxygenation,^13^ incapacitating free radicals that are generated in abundance during I/R.^14^ This significant reduction in hypoxic injury that melatonin exerts in the brain is an action not mediated through its receptors, but known to rely on the ability of the indoleamine as a radical scavenger.^15^

Little is known on the possible protective effects of melatonin in aged individuals affected by brain ischemia. In addition, the approach of previous studies has been to establish a subsequent reperfusion stage. However, during ischemia, the formation of colateral circulation that partially irrigates the ischemic lesion occurs. Thus, the lesion can be divided into umbral (necrotic neurons) and penumbral (damaged neurons) areas. The ability of the latter to be reperfused again is evident and melatonin, therefore, may exert its most beneficial effects on it. But, this remains to be clarified in aged individuals.

In previous investigations, we observed that melatonin prevented age-related apoptotic alterations and enhanced survival markers, as well as decreased the expression of proinflammatory cytokines in the aging hippocampus of old rats.^16,17^ Regarding I/R, we showed that melatonin was able to reduce alterations of the hepatic function in old rats subjected to transient liver vascular occlusion.^18^ In this study, the aim was to investigate the possible protective effect of melatonin on transcriptional markers involved in the inflammatory and apoptotic response secondary to ischemia induced by blockade of the right MCA in the hippocampus and cortex of aging male Wistar rats.

## Materials and Methods

### Animals

Male Wistar rats aged 2, 6, and 14 months (*n* = 5 per experimental group) were obtained from Harlan Ibérica (Barcelona, Spain) and maintained under standard conditions with a 12-h light–12-h dark photoperiod. Access to standard rodent chow (A04; Panlab, Barcelona, Spain) and water was permitted *ad libitum*. Animals were subjected to a model of ischemic brain injury consisting of the blockade of the right MCA. Groups of rats older than 14 months were not included in the study due to the dramatic mortality observed after that age during both the surgical procedure and in the immediate postoperative hours, the number of surviving animals therefore being very low. Control animals were not subjected to the surgical procedure or treated with either melatonin or ethanol.

The study was approved by the Ethics Committee of the Complutense University of Madrid (Madrid, Spain) in accordance with the European directive on the protection of animals used for scientific purposes (2010/63/EU).

### Surgical procedure

The surgery was carried out following a protocol previously described.^19,20^

Briefly, after premedication with fentanyl and medetomidine (0.3/0.3 mg/kg b.w.), animals were intubated and maintained under isoflurane anesthesia. Hair of the cervical ventral area was removed and, subsequently, rats were placed in supine position with the forelimbs open, the surgical field was prepared, and the area of intervention was sterilized. An incision was made in the midline from the laryngeal area up to 2 mm cranially to the xiphoid process and at that point it was lengthened 1.5 cm to the right. The right external carotid artery was exposed, carefully separated from the soft tissues of the area, and ligated with a silk 3/0 suture placed caudally to the dissected portion. A microvascular clip was then placed cranially to the ligature.

The carotid was opened in the middle point between the ligature and the microvascular clip with a 30-gauge needle. A 5/0 nylon filament was inserted up to the microvascular clip, which was removed to allow filament insertion into its final position. After assessing that the portion of filament introduced into the carotid artery was correctly positioned, a ligature was placed cranially to the site where the filament had been inserted. This assured that the filament remained in its position and did not move by the internal pressure of the artery. Finally, the absence of vascular bleeding was checked and suture in layers (continuous subcutaneous -vicryl 3/0- and skin with interrupted stitches -vicryl or silk 3/0 3/0-) was performed.

Isoflurane supply was interrupted, anesthesia was reversed with atipamezole (0.3 mg/kg b.w.), and a broad-spectrum antibiotic (enrofloxacin injectable solution) and an opioid analgesic (buprenorphine) were supplied. Analgesia was repeated at 12 and 24 h after the surgery. Throughout the surgical procedure and in the postsurgical recovery, the animals were placed on a heating blanket.

### Administration of melatonin

Melatonin (Actafarma, Madrid, Spain) was dissolved in absolute ethanol and then diluted with water to a final concentration of 0.1% and added to the drinking water at a dose allowing the supply of 10 mg/kg b.w./day in 25–30 mL. The dose of 10 mg/kg b.w. melatonin has been extensively used in scientific literature and it corresponds approximately to a dose of 1.62 mg/kg b.w. in humans, that is, around 100 mg/day for a 60 kg person.^21^ This dose in humans has been demonstrated to be safe. In fact, the daily administration to adults of doses up to 300 mg of melatonin does not produce significant adverse reactions.^22^ Moreover, follow-up studies of more than 10 years have reported no side effects.^23^

Fourteen-month-old animals (treated group) started the supply of this melatonin solution 24 h before the surgical procedure and during 24 h or 7 days of posttreatment period until sacrifice. Drinking water with 0.1% ethanol only was available for rats aged 2 and 6 months, as well as for the nontreated 14-month-old group. After both treatment periods, animals were sacrificed by decapitation and hippocampus and cortex of both ipsilateral and contralateral brain hemispheres to the ischemic lesion were carefully dissected. Tissues were immediately frozen in liquid nitrogen and kept at −80°C until analyzed.

### RNA isolation and reverse transcription–polymerase chain reaction

mRNA expression of interleukin-1 beta (IL-1β), tumor necrosis factor alpha (TNF-α), Bcl-2-associated death promoter (BAD), Bcl-2-associated X protein (BAX), glial fibrillary acidic protein (GFAP), B-cell lymphoma 2 (Bcl-2), and sirtuin 1 was measured by means of reverse transcription–polymerase chain reaction (RT-PCR).

RNA was isolated from hippocampus and cortex samples of rats using the TRI Reagent Kit (Molecular Research Center, Inc., Cincinnati, OH), following the manufacturer's protocol. The purity of the RNA was estimated with 1.5% agarose gel electrophoresis, and the RNA concentrations were determined with spectrophotometry (260 nm). Reverse transcription of 2 μg of RNA for cDNA synthesis was performed using the Reverse Transcription System (Promega, Madison, WI) and a pd(N)6 random hexamer. RT-PCR was performed using an Applied Biosystems 7300 apparatus with the SYBR Green PCR Master Mix (Applied Biosystems, Warrington, United Kingdom) and 300 nM concentrations of specific primers (Table 1).

**Table 1.** **Primers Used in Real-Time Polymerase Chain Reaction Experiments**

	Primers	Sequence (5′–3′)
18S	Forward	GGTGCATGGCCGTTCTTA
	Reverse	TCGTTCGTTATCGGAATTAACC
IL-1β	Forward	TGTGATGAAAGACGGCACAC
	Reverse	CTTCTTCTTTGGGTATTGTTTGG
TNF-α	Forward	ATGAGAAGTTCCCAAATGGC
	Reverse	CTCCACTTGGTGGTTTGCTA
BAD	Forward	GCCCTAGGCTTGAGGAAGTC
	Reverse	CAAACTCTGGGATCTGGAACA
BAX	Forward	GTGAGCGGCTGCTTGTCT
	Reverse	GGTCCCGAAGTAGGAGAGGA
GFAP	Forward	ACAGACTTTCTCCAACCTCCAG
	Reverse	CCTTCTGACACGGATTTGGT
Bcl-2	Forward	CAGGTATGCACCCAGAGTGA
	Reverse	GTCTCTGAAGACGCTGCTCA
Sirtuin 1	Forward	TCGTGGAGACATTTTTAATCAGG
	Reverse	GCTTCATGATGGCAAGTGG

18S was used as a housekeeping gene to compare the samples.

IL-1β, interleukin-1 beta; TNF-α, tumor necrosis factor alpha; BAD, Bcl-2-associated death promoter; BAX, Bcl-2-associated X protein; GFAP, glial fibrillary acidic protein; Bcl-2, B-cell lymphoma 2.

RT-PCR amplifications were performed as follows: 50°C for 2 min, 95°C for 10 min, followed by 40 cycles of 95°C for 15 sec, 60°C for 1 min, 95°C for 15 sec, 60°C for 30 sec, and 95°C for 15 sec. For the normalization of cDNA loading in the PCR, the amplification of 18S ribosomal RNA for every sample was used. Relative changes in the gene expression were calculated using the 2^−ΔΔCT^ method.

### Statistical analyses

Data are expressed as mean ± standard error of the mean of the number of determinations carried out in duplicate. The results were analyzed by ANOVA followed by Fisher's test. Statistical analyses were carried out using the SPSS 14.0 computer program (SPSS, Chicago, IL). The level of statistical significance was established at *p* < 0.05.

## Results

**FIG. 1.**
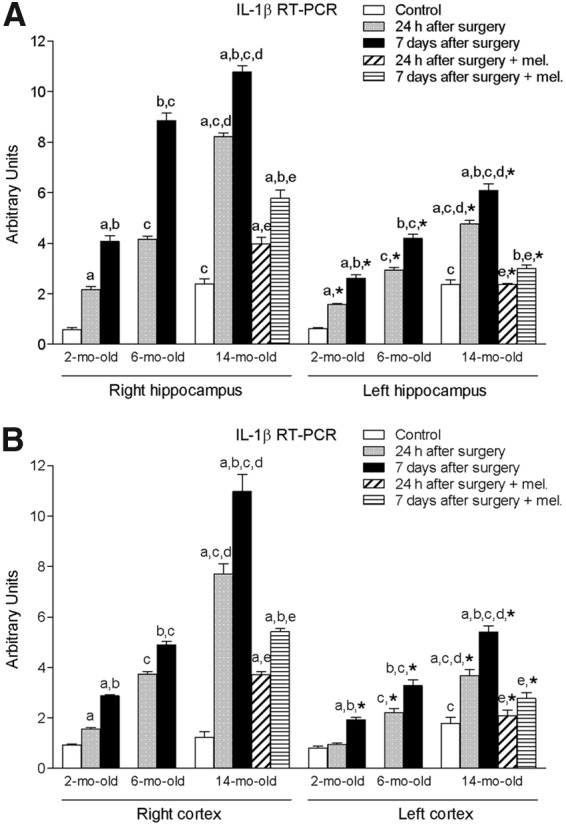
mRNA expression of interleukin-1 beta (IL-1β) in right and left hippocampus **(A)** and cortex **(B)** in nontreated rats aged 2 and 6 months and nontreated and melatonin-treated rats aged 14 months in control conditions and after 24 h or 7 days of being subjected to middle cerebral artery blockade. Each value represents the mean ± standard error of the mean of five determinations performed in duplicate. ^a^*p* < 0.05 values obtained in the control group; ^b^*p* < 0.05 values obtained at 24 h; ^c^*p* < 0.05 values obtained in the 2-month-old group; ^d^*p* < 0.05 values obtained in the 6-month-old group; ^e^*p* < 0.05 values obtained in their respective nontreated group; *p < 0.05 values obtained in the corresponding contralateral tissue.

The MCA blockade resulted in a significant increase (*p* < 0.05) of right hippocampus and cortex IL-1β expression after both 24 h and 7 days of procedure in the 2-month-old group compared to the control animals, with the 7-day values being significantly higher (*p* < 0.05) than those obtained 24 h after the surgery (Fig. 1A, B). A similar pattern was observed in the 6- and 14-month-old groups, but with age-related increasing values. The IL-1β mRNA levels reached in the 6-month-old rats were significantly augmented (*p* < 0.05) in comparison with their respective 2-month-old groups. Similarly, an additional significant elevation (*p* < 0.05) in the IL-1β expression was shown in the 14-month-old animals compared to the other two groups of age.

Treatment with melatonin decreased significantly the values of this proinflammatory cytokine (*p* < 0.05), although its levels after 7 days of procedure were still significantly higher when compared to those measured 24 h after it (*p* < 0.05).

As in the case of the right hippocampus and cortex, the procedure also provoked a significant rise in the IL-1β expression of left hippocampus and cortex (*p* < 0.05) with the values being higher with increasing age and days after surgery (Fig. 1A, B). Nevertheless, these values were significantly lower (*p* < 0.05) than those quantified in their respective right tissues. Again, a significant reduction (*p* < 0.05) in mRNA cytokine levels was observed in the melatonin-treated animals.

**FIG. 2.**
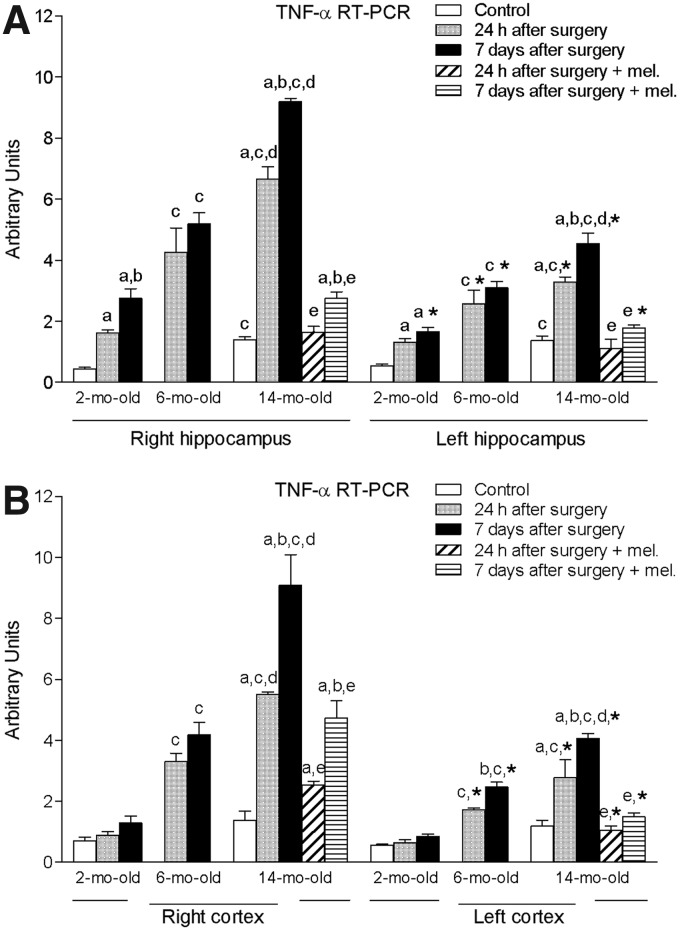
mRNA expression of tumor necrosis factor alpha (TNF-α) in right and left hippocampus **(A)** and cortex **(B)** in nontreated rats aged 2 and 6 months and nontreated and melatonin-treated rats aged 14 months in control conditions and after 24 h or 7 days of being subjected to middle cerebral artery blockade. Each value represents the mean ± standard error of the mean of five determinations performed in duplicate. ^a^*p* < 0.05 values obtained in the control group; ^b^*p* < 0.05 values obtained at 24 h; ^c^*p* < 0.05 values obtained in the 2-month-old group; ^d^*p* < 0.05 values obtained in the 6-month-old group; ^e^*p* < 0.05 values obtained in their respective nontreated group; *p < 0.05 values obtained in the corresponding contralateral tissue.

Regarding the right and left hippocampal levels of TNF-α, ischemic brain injury induced by the surgical procedure resulted in a significant increase (*p* < 0.05) of this cytokine in the three age groups (Fig. 2A). However, again the magnitude of this increase was significantly lower (*p* < 0.05) in the left area. In addition, and similar to what was shown for IL-1β, both the 6- and 14-month-old groups exhibited significantly higher levels of TNF-α (*p* < 0.05) compared to those reached in the 2-month-old animals. Fourteen-month-old values were also significantly elevated (*p* < 0.05) in comparison to the levels of the 6-month-old group. This was also observed in both right and left cortex, with the exception that no significant increase in the 2-month-old animals occurred (Fig. 2B).

Melatonin treatment was also able to reduce significantly (*p* < 0.05) the values of mRNA TNF-α in both hippocampus and cortex of the 14-month-old rats.

**FIG. 3.**
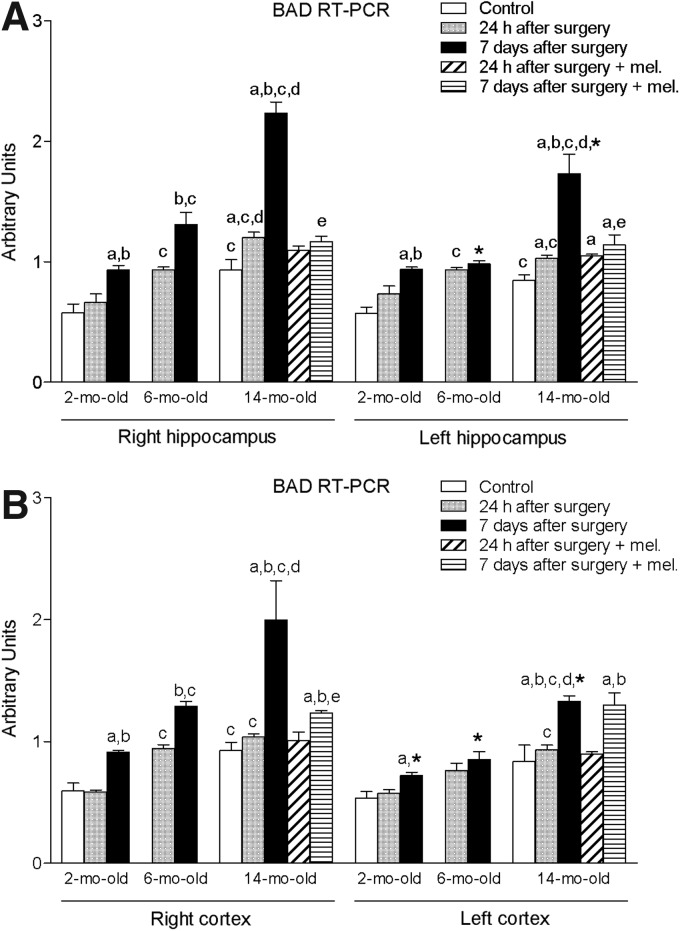
mRNA expression of Bcl-2-associated death promoter (BAD) in right and left hippocampus **(A)** and cortex **(B)** in nontreated rats aged 2 and 6 months and nontreated and melatonin-treated rats aged 14 months in control conditions and after 24 h or 7 days of being subjected to middle cerebral artery blockade. Each value represents the mean ± standard error of the mean of five determinations performed in duplicate. ^a^*p* < 0.05 values obtained in the control group; ^b^*p* < 0.05 values obtained at 24 h; ^c^*p* < 0.05 values obtained in the 2-month-old group; ^d^*p* < 0.05 values obtained in the 6-month-old group; ^e^*p* < 0.05 values obtained in their respective nontreated group; *p < 0.05 values obtained in the corresponding contralateral tissue.

**FIG. 4.**
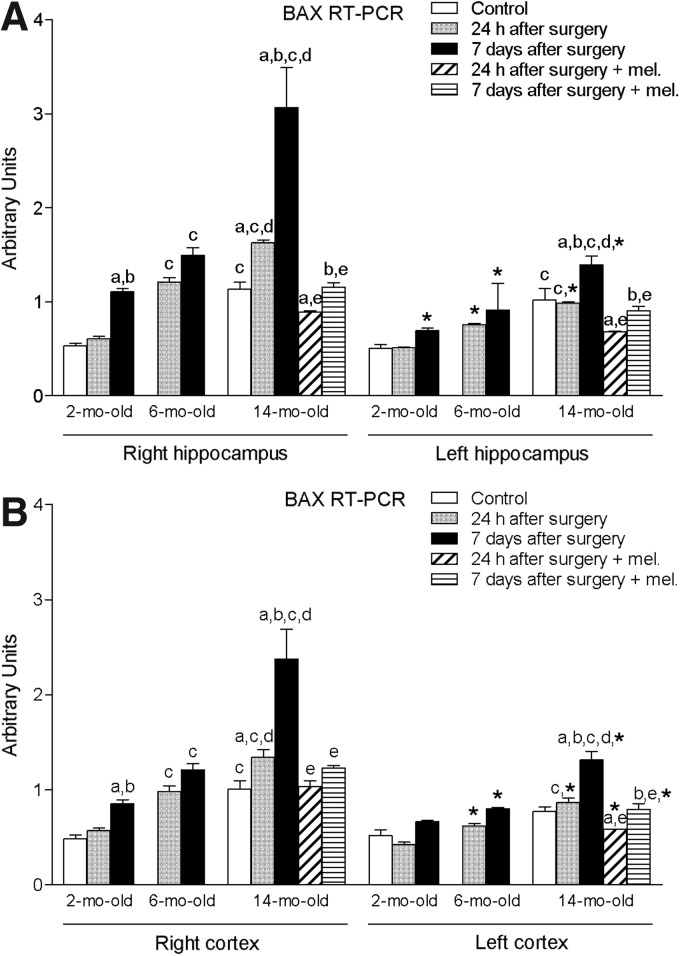
mRNA expression of Bcl-2-associated X protein (BAX) in right and left hippocampus **(A)** and cortex **(B)** in nontreated rats aged 2 and 6 months and nontreated and melatonin-treated rats aged 14 months in control conditions and after 24 h or 7 days of being subjected to middle cerebral artery blockade. Each value represents the mean ± standard error of the mean of five determinations performed in duplicate. ^a^*p* < 0.05 values obtained in the control group; ^b^*p* < 0.05 values obtained at 24 h; ^c^*p* < 0.05 values obtained in the 2-month-old group; ^d^*p* < 0.05 values obtained in the 6-month-old group; ^e^*p* < 0.05 values obtained in their respective nontreated group; *p < 0.05 values obtained in the corresponding contralateral tissue.

**FIG. 5.**
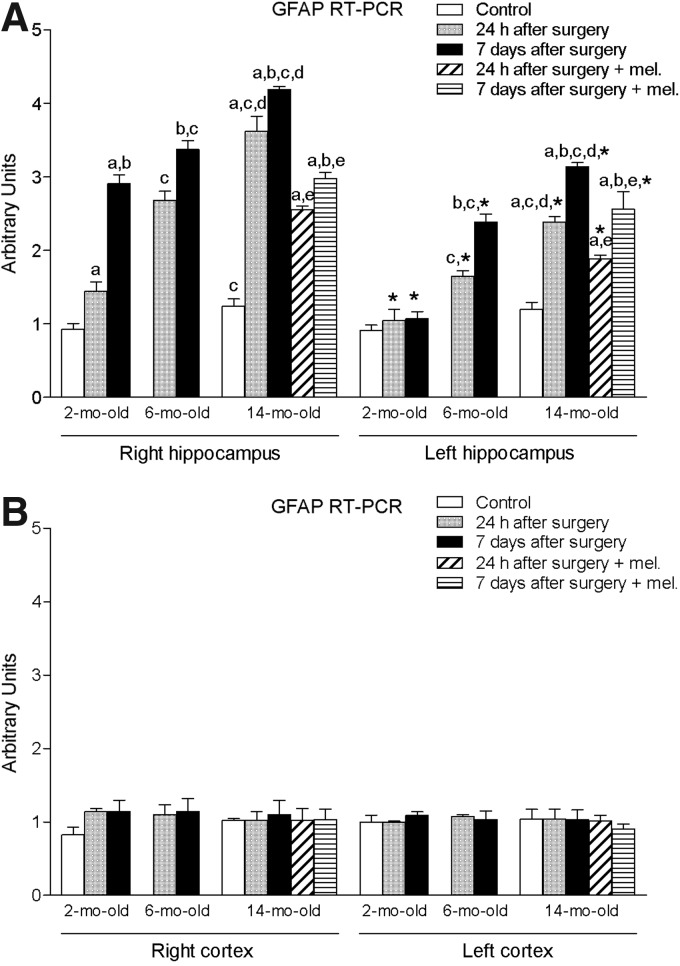
mRNA expression of glial fibrillary acidic protein (GFAP) in right and left hippocampus **(A)** and cortex **(B)** in nontreated rats aged 2 and 6 months and nontreated and melatonin-treated rats aged 14 months in control conditions and after 24 h or 7 days of being subjected to middle cerebral artery blockade. Each value represents the mean ± standard error of the mean of five determinations performed in duplicate. ^a^*p* < 0.05 values obtained in the control group; ^b^*p* < 0.05 values obtained at 24 h; ^c^*p* < 0.05 values obtained in the 2-month-old group; ^d^*p* < 0.05 values obtained in the 6-month-old group; ^e^*p* < 0.05 values obtained in their respective nontreated group; *p < 0.05 values obtained in the corresponding contralateral tissue.

Ischemia induced by MCA obstruction augmented significantly (*p* < 0.05) the right and left hippocampal levels of proapoptotic factors BAD and BAX (Figs. 3A and 4A). The levels of GFAP in this tissue were also significantly enhanced (*p* < 0.05) (Fig. 5A). As shown for the proinflammatory cytokines IL-1β and TNF-α, these increases were time- and age-dependent, with the highest values being obtained in the group of animals aged 14 months and after 7 days of the procedure. This was also the case for both right and left cortex (Figs. 3B and 4B), with the exception of GFAP, where no significant changes were observed (Fig. 5B). In general, ipsilateral values were also significantly higher (*p* < 0.05) than those quantified in the contralateral samples.

The afore-mentioned elevations were not seen in melatonin-treated animals.

**FIG. 6.**
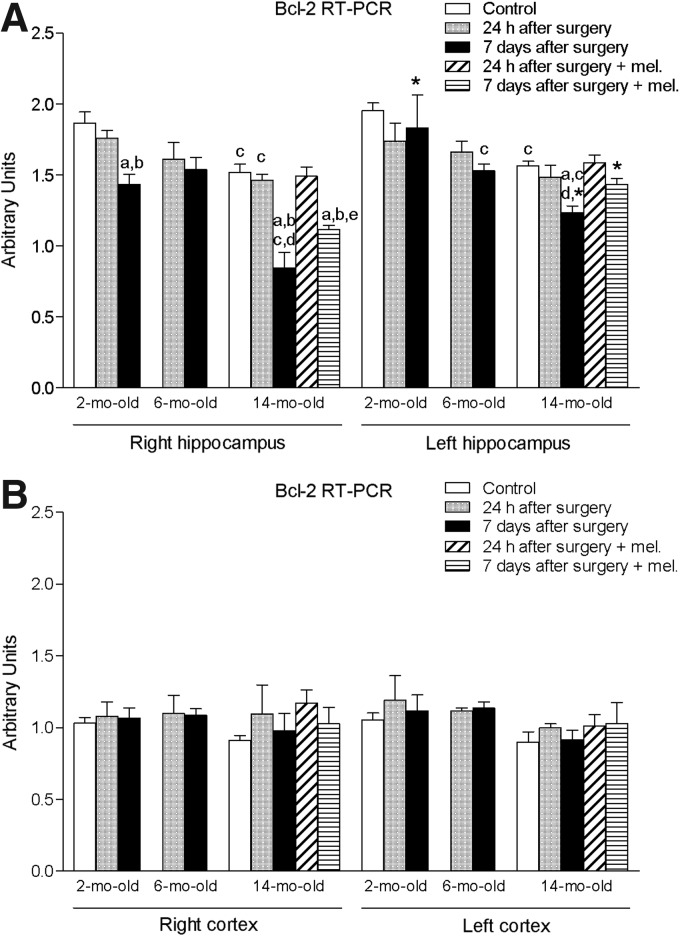
mRNA expression of B-cell lymphoma 2 (Bcl-2) in right and left hippocampus **(A)** and cortex **(B)** in nontreated rats aged 2 and 6 months and nontreated and melatonin-treated rats aged 14 months in control conditions and after 24 h or 7 days of being subjected to middle cerebral artery blockade. Each value represents the mean ± standard error of the mean of five determinations performed in duplicate. ^a^*p* < 0.05 values obtained in the control group; ^b^*p* < 0.05 values obtained at 24 h; ^c^*p* < 0.05 values obtained in the 2-month-old group; ^d^*p* < 0.05 values obtained in the 6-month-old group; ^e^*p* < 0.05 values obtained in their respective nontreated group; *p < 0.05 values obtained in the corresponding contralateral tissue.

The levels of antiapoptotic protein Bcl-2 experienced a significant decrease (*p* < 0.05) in the 14-month-old animals after 7 days of the procedure in both right and left hippocampus (Fig. 6A). Treatment with melatonin caused a significant elevation (*p* < 0.05) in the mRNA levels of this molecule in the right hippocampus. No significant changes were observed in right and left cortical mRNA Bcl-2 expression (Fig. 6B).

**FIG. 7.**
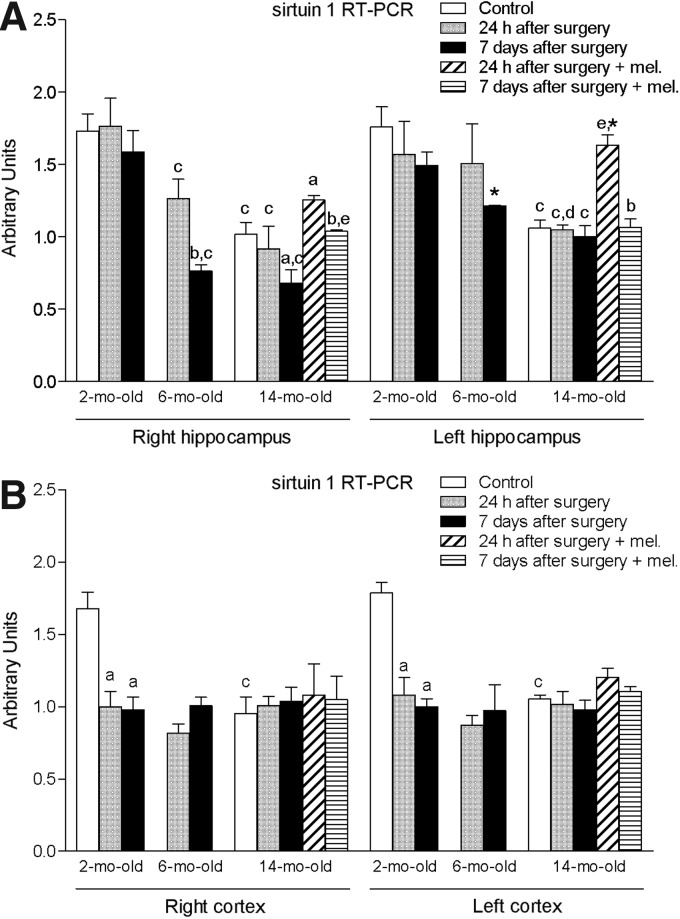
mRNA expression of sirtuin 1 in right and left hippocampus **(A)** and cortex **(B)** in nontreated rats aged 2 and 6 months and nontreated and melatonin-treated rats aged 14 months in control conditions and after 24 h or 7 days of being subjected to middle cerebral artery blockade. Each value represents the mean ± standard error of the mean of five determinations performed in duplicate. ^a^*p* < 0.05 values obtained in the control group; ^b^*p* < 0.05 values obtained at 24 h; ^c^*p* < 0.05 values obtained in the 2-month-old group; ^d^*p* < 0.05 values obtained in the 6-month-old group; ^e^*p* < 0.05 values obtained in their respective nontreated group; *p < 0.05 values obtained in the corresponding contralateral tissue.

Finally, ischemia induced by MCA blockade provoked a significant reduction (*p* < 0.05) in the right and left hippocampal expression of sirtuin 1, most notably observed in the 14-month-old group (Fig. 7A). The administration of melatonin was able to attenuate, in part, this decrease, with significantly higher values (*p* < 0.05) in the mRNA expression of sirtuin 1 in the right and left hippocampus after 7 days or 24 h of ischemic injury, respectively. With regard to the cortex, the procedure was only able to reduce significantly (*p* < 0.05) the mRNA levels of sirtuin 1 in the 2-month-old animals at both times evaluated (Fig. 7B). However, the control values of the old animals were significantly lower (*p* < 0.05) than those measured in the control young group.

## Discussion

Stroke is a major health problem with a significant impact on the affected individuals and the whole community. Neural ischemia occurs locally (focal ischemia) when a single blood vessel is involved, or the ischemic event can involve the entire brain (global ischemia), for example, during the transient discontinued beating of the heart. Regardless of whether the neural lesion is focal or global, the mechanisms of free radical-mediated neurological damage have many common features.

The most frequently used model to test the ability of melatonin to curtail free radical damage that occurs of a result of ischemia is MCA occlusion.^9^ Since the surgical procedure also involves the occlusion of the primitive carotid artery, the damage is not only circumscribed to the ipsilateral side but also to the contralateral, as has been described earlier.^24^ In addition, the lesion is not only present in the hippocampus but also in the cortex.^25^

Increasing data from experimental studies have shown that melatonin treatment may improve the outcome of patients suffering from acute focal or global cerebral ischemia.^8,26^ In addition, it is known that there is a gradual reduction in circulating melatonin concentrations with increasing age, leading some to speculate that its loss contributes to the aging process. This fact may be the reason why more marked ischemia-induced deleterious effects are seen in aged animals. It also may explain the numerous beneficial effects that supplemental melatonin has been shown to display in terms of seemingly forestalling some signs of age-related organ deterioration, including the brain.^27^

To our knowledge, however, these possible protective effects of melatonin on stroke-induced ischemia in aged animals have not been addressed yet.

A very large number of studies have documented the ability of melatonin to detoxify harmful reactants and reduce molecular damage.^12^ Regarding aging, we have consistently reported that melatonin was able to revert age-related augmentation of inflammation, oxidative stress, and apoptosis markers, as well as to prevent the age-related diminution of anti-inflammatory and antiapoptotic mediators in the heart,^28,29^ pancreas,^30^ or liver.^18^ In the brain, we showed previously that melatonin treatment was able to decrease mRNA levels of BAD, BAX, and Bcl-2 among other proapoptotic markers.^16,17^ Similar results have been obtained for IL-1β or TNF-α.^17,31^

In the present study, expression of TNF-α, IL-1β, BAD, and BAX increased significantly in both ipsilateral and contralateral hippocampus and cortex after occlusion of the MCA. These effects were noticeably more deleterious and aggravated with increasing age, that is, 6- and 14-month-old groups exhibited significantly higher levels compared to those reached in the 2-month-old animals, with the highest values being obtained in the group of animals aged 14 months. Melatonin-treated animals experienced a significant decrease in TNF-α, IL-1β, BAD, and BAX in the ischemic ipsilateral and contralateral areas of both right and left hippocampus and cortex.

Melatonin's protective actions have been attributed to the ability of the indoleamine to scavenge free radicals that are generated in abundance during I/R.^9,14^ Likewise, melatonin may aid in reducing neural damage in ischemia-induced injury by limiting apoptosis. Indeed, it has been documented that melatonin reduced infarct volume and apoptosis that resulted from cerebral I/R, preventing the injury-induced reduction in Akt activation and drop in phosphorylation of mTOR and p70S6 kinase, as well as the subsequent decrease in S6 phosphorylation.^32^ Our results suggest that melatonin treatment mediates neuroprotection against ischemic injury at least partly by inhibition of the consequential inflammatory response, aiding to limit tissue destruction in the affected brain region.^33^

Chemical and mechanical insults to the brain cause permanent changes with astrocytes responding through a variety of reactions. Reactive astrocytes exhibit extensive synthesis of GFAP, hypertrophy, proliferation, and changes in energy metabolism.^34^ The rapid synthesis of GFAP is a highly characteristic and well-documented phenomenon and it responds quickly to physical or metabolic injury, including oxidative stress.^35^

Prolonged light exposure, which reduces endogenous melatonin production, has been found to cause an elevation in GFAP levels in the brain, while melatonin administration was able to decrease it.^36^ In our model, where the effect of an ischemic lesion was evaluated together with the age-related physiological reduction of the indoleamine, a similar observation was noted in the hippocampus. This supports the hypothesis that the increased expression of GFAP in advanced age may be at least partially the result of the decrease in melatonin levels.

Other researchers have reported that melatonin treatment was not able to influence the I/R-induced GFAP response, suggesting that it may not mediate neuroprotective actions following I/R through astrocytic mechanisms.^37^ Since these results were obtained in putamen, parietal cortex, and insular cortex, and not in hippocampus, as was our case, further investigation is needed to elucidate the role of melatonin on astrocytic response/survival following an I/R injury in the brain.

A number of studies have unequivocally supported the idea of sirtuins having therapeutic potential in neurodegenerative diseases such as stroke or an ischemic brain injury.^38^ Sirtuin 1 has been also related to apoptosis reduction, whereas its inactivation seems to promote translocation of BAX from the cytosol to mitochondria.^39^ In previous investigations, we showed that the expression of sirtuin 1 was reduced in the dentate gyrus of old rats and its expression was increased after melatonin treatment.^16^ In this study, we also observed that melatonin was able to counteract the hippocampal decrease of sirtuin 1 due to blockade of the MCA. This supports previous reports suggesting that melatonin was able to increase the deacetylation of sirtuin 1 targets and has a stimulatory effect on the sirtuin 1 pathway.^40^

Considering stroke as the leading cause of disability, the ageing of the population and high incidence of this cerebrovascular insult among the elderly, the importance of finding primary and secondary prevention interventions for this group becomes essential. In our study, treatment with melatonin was able to decrease significantly the hippocampal and cortex expression of proinflammatory IL-1β and TNF-α and proapoptotic BAX and BAD markers, as well as reduce the mRNA levels of GFAP and elevate sirtuin 1 in ageing rats subjected to ischemic lesion induced by blockade of the MCA. In light of the present results, melatonin appears as a valuable therapeutic agent that may protect the elderly from the damaging effects of stroke, with potential actions in preventing the extension of the lesion.

## Abbreviations Used

BAD: Bcl-2-associated death promoter
BAX: Bcl-2-associated X protein
Bcl-2: B-cell lymphoma 2
GFAP: glial fibrillary acidic protein
I/R: ischemia and reperfusion
IL-1β: interleukin-1 beta
MCA: middle cerebral artery
mTOR: mammalian target of rapamycin
RT-PCR: reverse transcription–polymerase chain reaction
TNF-α: tumor necrosis factor alpha
